# **AHSCs as Health Policy Transfer: Some Emergent Evidence From Australia** Comment on "Academic Health Science Centres as Vehicles for Knowledge Mobilisation in Australia? A Qualitative Study"

**DOI:** 10.34172/ijhpm.2022.6284

**Published:** 2022-02-12

**Authors:** Ewan Ferlie

**Affiliations:** King’s Business School, King’s College London, London, UK.

**Keywords:** Health Sciences Centres, Australian Healthcare, Knowledge Translation, Knowledge Mobilization, Health Policy Transfer, Governance

## Abstract

This commentary discusses Edelman et al 2020’s recent exploratory study of the early development of 4 Academic Health Services Centres (AHSCs) in Australia. AHSCs were originally invented in the United States, but have then diffused to the United Kingdom and Canada over the last decade or so and now to Australia so they are a good example of health policy transfer. They are dedicated to advancing more speedy knowledge translation (KT)/mobilization (‘from bench to bedside’) and also the more effective commercialization of scientific inventions. The commentary argues some interesting if preliminary findings are identified in their study. Its limitations will also be considered. Finally, suggestions for future research are made, including more cross national and comparative studies.

 This commentary discusses Edelman et al^1^ 2020’s recent exploratory study of the early development of 4 Academic Health Services Centres (AHSCs) in Australia. AHSCs represent a good example of international policy transfer in the designing and diffusing of a novel organizational form in healthcare. The spread of AHSCs reflects continuing health policy interest in supporting more effective knowledge mobilization (KM)^2-4^ and also commercialization activity. AHSCs were originally invented in the United States, but then have moved to the United Kingdom^4^ and Canada^5^ over the last decade or so and now to Australia.

 AHSCs represent a new organizational form bringing together three basic functions of clinical practice, the teaching of medical students, and scientific research. The AHSC framework hopes to accelerate the flow of new clinical knowledge ‘from bench to bedside’ and also promote quicker commercialisation of scientific discoveries, thus enhancing the contribution of the healthcare sector to wealth and economic growth. The AHSC settings hope to accomplish speedier mobilization of new knowledge from an expanding basic R and D function^3^ and thereby reduce delays between new scientific discoveries and embedding them into clinical practice and the medical school curriculum. Although there have been some attempts to establish a range of partnerships with other healthcare settings (more evident in some cases than others), the UK AHSCs are generally concentrated in a core of elite academic and generally acute sector orientated settings, all with exceptionally strong basic research profiles. Indeed, that was the rationale for their selection. Primary and community health services and social care settings have so far been less represented.

 Edelman et al^1^ examine the early development of 4 AHSCs in Australia and explore how they are ‘organizing for impact’ at this initial stage. The authors conclude (p. 5): ‘the findings of this study illustrate that AHSCs in Australia are in relatively early stages of development, with different AHSCs following different pathways.’ So local context, history and variation was important. Their study took place at a very early stage so it was as yet impossible to assess their full impact (and indeed a prior methodological conversation was needed about how assess impact robustly). Many different and not always aligned indicators could assess success. But are there some themes which emerge across the four AHSCs, even at this early stage?

## Organizational Governance

 There was much attention found to designing governance arrangements (as in some earlier UK case study work^6^) in the sites with concern to make sure all partners had adequate representation (but sometimes at the cost of creating unwieldy mechanisms). In the UK case, AHSCs are governed as confederations rather than as a single vertically integrated organization. The partners (National Health Service [NHS] Trusts and Universities) retain their own Boards and governance systems, so that the organization of the AHSC here takes the form of a relatively small cross organizational team laid on top of large and complex sovereign organizations.

 There was intriguing internal variation in governance structures found in the 4 Australian AHSCs. One AHSC was an integrated organization, following the more US model. Three, however, formed multi organizational governance structures and were focussed on working together at scale across organizational boundaries. This model added in an additional dimension of complexity. We can ask some questions: are these governance arrangements always cooperative in practice or are there some underlying tensions, given the scale and importance of these confederations? How is legal liability distributed in these complex settings? Does the overall AHSC Board have real powers or do these remain with the Boards of the constituent organizations which may remain legally sovereign? How is budgetary authority constituted and who is financially liable if the AHSC goes into financial crisis?

 The authors^1^ concluded that ‘soft’ features such as: shared values, aligned expectations and high trust were important in making collaboration happen and in overcoming a possible legacy of inter organizational competition. So local organization climates, culture and networking practices are all likely to be important capabilities as well as formal governance arrangements.

## AHSCs in Less Acute Sector Dominated and More Distributed Settings

 Two of the AHSCs were urban based and located in large metropolitan cities, which is a conventional profile. Yet two (and this is novel) were set in rural and remote geographies and importantly with more a community and population focus than conventional and more clinically orientated acute sector settings. It may be that the type of knowledge preferred here is broader than in acute sector settings.

 It can be asked: what does high quality research based knowledge mean here? What designs, methods and data sources are used in such research and are they different from the emphasis on Randomised Control Trials often apparent in the acute sector? Do primary, community and social care based forms of evidence here have a higher profile? How evident is public health and population based knowledge and evidence? Furthermore, how can a ‘good’ research knowledge base be mobilised effectively in these more geographically distributed settings with fewer large core institutions which might be expected to have some internal knowledge processing capacity? It would be interesting to track the research output from such distributed settings over time – starting now – and attempt to assess its long-term quality and impact (although this is not an easy task, as mentioned above). Are some distributed settings more productive in research terms and if so, how and why? This is also an important strategic issue for research leaders in these settings and more broadly in the wider national R and D function which they may wish to consider now so as to develop such a long-term assessment framework.

## A Limited Shift From Knowledge Translation to Knowledge Mobilization

 The KM theme is again highlighted in the paper, as in earlier UK and international work.^2,3^ Recent social science informed work has moved away from a reliance on formal knowledge management systems or linear models of knowledge translation (KT) to emphasise more contextual, process and practice informed models of enacted KM (FitzGerald and Harvey^7^ explore such issues in a related English network based ‘research into practice’ setting; also see Swan et al’s edition^8^).

 However, respondents in the sites were found still to use the KT term (assuming a ‘push’ from researcher based knowledge) in preference to wider knowledge mobilisation. KT activity was still seen as at an early stage in the sites but strategies to progress KT (some of which were as yet aspirational) included: flagship projects; capacity building and working through clinical leaders. The finding that broader knowledge mobilisation activity as yet remained limited needs to be followed up as this may represent a major weakness. Also it can be asked: what projects are the AHSCs progressing in the commercialization domain which was a topic not really fully covered here?

## Changing Modes of Research Production?

 There was an intent expressed by some AHSC respondents to change the mode of research production away from a traditional and academically directed (Mode 1) form towards a more socially distributed or mode 2 mode^9^ and thereby help create more impactful research. Co-produced research and more research shaped by thematic priorities were mentioned as potential strategies to counteract what might otherwise be a succession of individual and academically dominated projects. Yet other respondents mentioned that major researchers remained powerful in the AHSCs. Mode 1 (traditional and academic dominated) research may be highly resilient in practice and reinforced by disciplinary based funding and publications conventions. So any transition to Mode 2 may be partial and contested (reinforcing an earlier analysis^10^of a sample of high performing research groups in the English healthcare system which found basic disciplines were still embedded). Undertaking co-produced research is not easy in practice and may require researchers to acquire additional skills. It might be interesting to look at papers from those settings (such as mental health and also local government) with more experience in such work. Public services organizations in certain countries – such as Scandinavia – also appear to have built up a level of experience in this domain so that there may be an international knowledge case to explore.

 Of course, there are other limitations to this exploratory study, as the authors themselves recognise. The number of interviews is small (15) and weighted to senior level respondents, so other important voices are less visible. The study is inductive and while clear themes are identified there is not so far an explicitly developed theoretical framework.

 However, this commentary has suggested that the following specific themes: AHSC governance; AHSCs operating in less urban settings; KM and translation processes; and the extent to which there are changing modes of research production all appear as interesting initial findings which could be usefully followed up. Future larger scale work should track these Australian sites over time as well as undertake more comparative analysis with other countries. An encouraging feature of this Australian study is that some investigators have experience of earlier UK AHSC related research. Hopefully, such mixed teams can help stimulate more international comparison and learning in future work.

 Given the potential importance of AHSCs as novel settings for KM; it is odd and disappointing (including in the United Kingdom) that we still know so little about them. It is the case that the AHSC literature is developing in some areas. For instance, Edelman et al^11^ provide a useful recent systematic review on how AHSCs can support equity in healthcare systems. But there is a need to undertake more longitudinal and theoretically informed case study work (Fischer et al^6^ represents an early single case example from London but more are needed) to build a comparative, international and publicly accessible knowledge base about the still developing AHSCs.

 Finally, it can also be remarked that the United Stattes, the United Kingdom, Canadian and here Australian cases all come from ‘first world’ settings, indeed from a mainly Anglophone group (with the important exception of Quebec). Are AHSCs diffusing to different health systems, for example in medium income countries with rapid economic growth in the Global South, or do they remain a Global North phenomenon?

## Ethical issues

 Not applicable.

## Competing interests

 Author declares that he worked with one of the Australian team, Michael Fischer, who led an earlier AHSC case study^6^ based in London.

## Author’s contribution

 EF is the single author of the paper.
